# Doxycycline prophylaxis for sexually transmitted infections prevention among men who have sex with men in China

**DOI:** 10.3389/fpubh.2025.1736363

**Published:** 2026-01-15

**Authors:** Yi Ding, Rong Su, Lishan Tian, Qiuhong Wu, Hailing Ye, Mingjing Xue, Silan Chen, Jun Yuan

**Affiliations:** 1 Shenzhen Nanshan Center for Chronic Disease Control, Shenzhen, China; 2 Department of Medical Statists, School of Public Health, Sun Yat-sen University, Guangzhou, China

**Keywords:** associated factors, doxycycline prophylaxis, men who have sex with men, sexually transmitted infections, utilization

## Abstract

**Background:**

Men who have sex with men (MSM) have a high prevalence of sexually transmitted infections (STIs). The efficacy of doxycycline prophylaxis in STIs prevention has been demonstrated. This study aimed to examine the utilization of doxycycline prophylaxis among MSM in China, providing evidence to inform future policies and guidelines.

**Methods:**

Between June and July 2025, a cross-sectional survey was conducted in China, using a convenience sampling method to recruit eligible MSM. Sociodemographic characteristics, behavioral information, and doxycycline prophylaxis utilization experience were collected from the participants. Logistic regression analysis was performed to identify factors associated with doxycycline prophylaxis utilization among MSM.

**Results:**

A total of 508 MSM were recruited, with a median age of 31.5 (28.0–64.0) years. The utilization proportion of doxycycline prophylaxis was 13.4% (68/508). Multivariate logistic regression analysis indicated that age >35 years (aOR = 0.393, 95%CI: 0.172–0.898), engagement in casual sex (aOR = 2.187, 95%CI: 1.045–4.578), engagement in commercial sex (aOR = 3.986, 95%CI: 1.622–9.794) and HIV PrEP/PEP experience (aOR = 20.220, 95%CI: 9.061–45.123) were significantly associated with doxycycline prophylaxis use.

**Conclusions:**

As an effective strategy for STIs prevention, doxycycline prophylaxis use among MSM in China remains at a relatively low level. Age and sexual behaviors are associated with its use. Future efforts should focus on strengthening STIs-related education, enhancing MSM's risks awareness, and promoting the appropriate use of doxycycline prophylaxis for STIs prevention within this population.

## Introduction

Sexually transmitted infections (STIs) are among the most prevalent infectious diseases worldwide, posing significant threats to public health and individual well-being. According to the World Health Organization (WHO), more than one million people acquire STIs every day (1). Most STIs are asymptomatic (2). If left untreated, they can lead to severe complications beyond the immediate effects of infection, including neurological and cardiovascular diseases, infertility, ectopic pregnancy, and stillbirths. Moreover, infections such as herpes, gonorrhoeae and syphilis can increase the risk of HIV acquisition (3).

STIs spread mainly through unprotected sexual contact. Men who have sex with men (MSM) remain a key population for STIs due to behaviors, such as unprotected anal sex, multiple sexual partners, casual sex, and group sex (4). Among Chinese MSM, *Chlamydia trachomatis, Neisseria gonorrhoeae*, and *Treponema pallidum* are the most commonly reported pathogens. Recent data from Guangdong Province indicated a rising prevalence of *Chlamydia trachomatis* infection among Chinese MSM, increasing from 13.8% in 2018 to 26.4% in 2022 (5). The prevalence of *Neisseria gonorrhoeae* infection was about 2.5% (6), while *Treponema pallidum* infection among MSM continues to be a major public health concern, particularly because of its frequent co-infection with HIV (7).

Current strategies for STIs prevention in China primarily focus on condom promotion and regular testing. Studies have shown that condom promotion programs have achieved moderate success, leading to a notable increase in condom use (8, 9). However, due to the pursuit of sexual pleasure (10), consistent condom use among MSM remains low, thereby limiting its protective effectiveness. Regular testing is a key strategy for identifying STIs, yet the overall STIs testing rate among Chinese MSM remains low. Notably, since syphilis screening is often coupled with HIV testing (such as HIV/syphilis dual test kits), syphilis testing rates among MSM tend to be higher than those for other STIs. Overall, traditional STIs prevention measures remain inadequate to curb the persistent STIs epidemic. In the absence of effective vaccines, researchers and policymakers are actively exploring pharmacological approaches to prevent STIs.

Doxycycline, a broad-spectrum tetracycline antibiotic, is widely recommended for the treatment of chlamydia and syphilis due to its safety, affordability, and minimal drug interactions. In recent years, numerous studies have examined the efficacy of doxycycline prophylaxis in preventing STIs (11, 12). Findings indicated that both daily uptake (doxy-PrEP) and post-exposure intake within 24–72 h after unprotected sexual contact (doxy-PEP) can significantly reduce STIs incidence. Recognizing its preventive potential, the US Centers for Disease Control and Prevention and the British Association for Sexual Health and HIV have issued guidelines recommending doxy-PEP for bacterial STIs prevention among high-risk populations, including MSM (13, 14). Similarly, Germany and Australia have released position statements supporting the prophylactic use of doxycycline for STIs prevention (15, 16).

Despite increasing international evidence supporting doxycycline prophylaxis for STIs prevention, data from China remain limited. Therefore, we conducted a cross-sectional survey among Chinese MSM to assess the current use of doxycycline prophylaxis, aiming to provide evidence for STIs prevention in China.

## Methods

### Study design and participants

From June to July 2025, a web-based cross-sectional survey was conducted among MSM in China. The sample size was calculated using the following formula: *N* = $\frac{Z_{1-\alpha /2}^{2}\times pq}{d^{2}}$, *p* = 24.5% (17). This calculation indicated a required sample size of 296. Eligible participants aged 18 years or older were recruited using a convenience sampling method. Specifically, recruitment was conducted through the STD clinic of Shenzhen Nanshan Center for Chronic Disease Control and its online platform. The participants were drawn from various regions across China. Prior to enrollment, all participants were informed of the study's background and objectives, and they had the right to decline participation. After completing the questionnaire, participants were encouraged to share the survey with their peers to facilitate the recruitment of additional eligible individuals.

To promote participation, all respondents received small incentives, such as condoms. To ensure data quality, all participants were required to connect with the research team via the online platform, enabling researchers to address any issues arising during the survey process. The research team reviewed completed questionnaires daily and promptly verified and corrected any inconsistencies or missing responses.

### Measures

All data were collected through an online questionnaire. The following information was collected: sociodemographic characteristics (age, marital status, education, monthly income, sexual orientation and sexual role), sexual behavior in the last 6 month (consistent condom use, multiple sexual partners, casual sex, commercial sex, group sex), STIs testing, HIV PrEP/PEP experience and doxycycline prophylaxis utilization experience.

### Statistical analysis

Data management and statistical analysis were performed using R software (Version 4.5.1). Descriptive statistics were utilized to summarize participants' basic characteristics. Logistic regression models were employed to identify factors associated with doxycycline prophylaxis use among MSM, calculating odds ratio (OR) and 95% confidence intervals (CI). Variables with *P* < 0.2 in univariate analysis were included in the multivariate model. All statistical tests were two-sided, and *P* < 0.05 was considered statistically significant.

## Results

### Study population

A total of 508 MSM were recruited for the study, with a median age of 31.5 (28.0–64.0) years, predominantly aged ≤ 35 years, constituting 73.0% (371/508). Among the participants, 84.8% (431/508) were unmarried, 86.2% (438/508) held a bachelor's degree or below, 78.7% (400/508) reported a monthly income of < 15,000 RMB, and 80.9% (411/508) reported homosexual orientation. Regarding sexual roles, 30.3% (154/508) reported being an insertive partner, 19.9% (101/508) receptive, and 49.8% (253/508) engaged in both roles (Table 1).

**Table 1:** Sociodemographic characteristics of participants recruited in this study (*N* = 508).

**Characteristics**	***N***	**Percentage (%)**
**Age (years)**		
≤ 35	371	73.0
>35	137	27.0
**Marital status**		
Married/divorced/widowed	77	15.2
Unmarried	431	84.8
**Education**		
Bachelor or below	438	86.2
Master or above	70	13.8
**Monthly income (RMB)**		
< 15,000	400	78.7
≥15,000	108	21.3
**Sexual orientation**		
Heterosexual/bisexual/uncertain	97	19.1
Homosexual	411	80.9
**Sexual role**		
Inserted	154	30.3
Receptive	101	19.9
Both	253	49.8

Exchange rate: 1 USD ≈ 7.2 RMB.

### Doxycycline prophylaxis utilization and associated factors

Among the study participants, 68 individuals reported having ever used doxycycline prophylaxis for STIs prevention, corresponding to a utilization proportion of 13.4% (68/508). Of the doxycycline prophylaxis users, 85.3% (58/68) were aged ≤ 35 years, 89.7% (61/68) were married/divorced/widowed, 80.9% (55/68) had a bachelor's degree or lower, 70.6% (48/68) reported a monthly income of < 15,000 RMB, 75.0% (51/68) reported homosexual orientation, and 53.0% (36/68) played both inserted and receptive roles in sexual behavior. Among them, 51.5% (35/68) reported consistent condoms use during sex, 41.2% (28/68) had multiple sexual partners, 79.4% (54/68) engaged in casual sex, 23.5% (16/68) in commercial sex, 32.4% (22/68) in group sex, 64.7% (44/68) had undergone STIs testing, and 86.8% (59/68) had ever used PEP/PrEP for HIV prevention (Table 2).

**Table 2:** Logistic regression analysis of factors associated with doxycycline utilization.

**Characteristics**	**Total**	**Doxycycline utilization**	**Univariate analysis**		**Multivariate analysis**	
			**OR (95% CI)**	***P***	**aOR (95% CI)**	***P***
**Age (years)**						
≤ 35	371	58 (85.3%)	1.000		1.000	
>35	137	10 (14.7%)	**0.425 (0.211–0.857)**	**0.017**	**0.393 (0.172–0.898)**	**0.027**
**Marital status**						
Married/divorced/widowed	431	61 (89.7%)	1.000			
Unmarried	77	7 (10.3%)	0.607 (0.266–1.381)	0.234		
**Education**						
Bachelor or below	438	55 (80.9%)	1.000		1.000	
Master or above	70	13 (19.1%)	1.588 (0.816–3.089)	0.173	1.168 (0.505–2.700)	0.717
**Monthly income (RMB)**						
< 15000	400	48 (70.6%)	1.000		1.000	
≥15000	108	20 (29.4%)	1.667 (0.941–2.952)	0.080	0.918 (0.446–1.891)	0.816
**Sexual orientation**						
Heterosexual/bisexual/uncertain	97	17 (25.0%)	1.000		1.000	
Homosexual	411	51 (75.0%)	0.667 (0.366–1.215)	0.185	0.500 (0.241–1.038)	0.063
**Sexual role**						
Inserted	154	20 (29.4%)	1.000			
Receptive	101	12 (17.6%)	0.903 (0.421–1.940)	0.794		
Both	253	36 (53.0%)	1.112 (0.618–2.000)	0.724		
**Consistent condom use**						
No	206	33 (48.5%)	1.000		1.000	
Yes	302	35 (51.5%)	0.687 (0.412–1.147)	0.151	0.742 (0.401–1.374)	0.343
**Multiple sexual partners**						
No	347	40 (58.8%)	1.000		1.000	
Yes	161	28 (41.2%)	1.616 (0.957–2.719)	0.073	0.865 (0.454–1.647)	0.659
**Casual sex**						
No	206	14 (20.6%)	1.000		1.000	
Yes	302	54 (79.4%)	**2.986 (1.611–5.536)**	**< 0.001**	**2.187 (1.045–4.578)**	**0.038**
**Commercial sex**						
No	453	52 (76.5%)	1.000		1.000	
Yes	55	16 (23.5%)	**3.164 (1.652–6.058)**	**< 0.001**	**3.986 (1.622–9.794)**	**0.003**
**Group sex**						
No	410	46 (67.6%)	1.000		1.000	
Yes	98	22 (32.4%)	**2.291 (1.302–4.030)**	**0.004**	1.417 (0.675–2.974)	0.357
**STIs testing**						
No	214	24 (35.3%)	1.000			
Yes	294	44 (64.7%)	1.393 (0.816–2.372)	0.222		
**HIV PrEP/PEP experience**						
No	327	9 (13.2%)	1.000		1.000	
Yes	181	59 (86.8%)	**17.087 (8.220–35.521)**	**< 0.001**	**20.220 (9.061–45.123)**	**< 0.001**

Odds ratio was calculated using logistic regression models. Multivariate analysis included variables with *P* < 0.2 in univariate analysis. Exchange rate: 1 USD ≈ 7.2 RMB. Indicators with *p*-value < 0.05 are displayed in bold.

Univariate logistic regression analysis showed that age (OR = 0.425, 95%CI: 0.211–0.857), casual sex (OR = 2.986, 95%CI: 1.611–5.536), commercial sex (OR = 3.164, 95%CI: 1.652–6.058), group sex (OR = 2.291, 95%CI: 1.302–4.030), and HIV PrEP/PEP experience (OR = 17.087, 95%CI: 8.220–35.521) were significantly associated with doxycycline prophylaxis utilization. Multivariate analysis identified that age >35 years (aOR = 0.393, 95%CI: 0.172–0.898), engagement in casual sex (aOR = 2.187, 95% CI: 1.045–4.578) and commercial sex (aOR = 3.986, 95%CI: 1.622–9.794) in the last 6 months, and previous HIV PrEP/PEP use (aOR = 20.220, 95%CI: 9.061–45.123) remained significantly associated with doxycycline use (Table 2).

## Discussion

In this study, 13.4% of participants had used doxycycline prophylaxis for STIs prevention, a proportion lower than those reported in previous studies. A study conducted in Taiwan found that 24.5% of participants had ever used doxy-PrEP (17). In Germany, 23% of participants had used doxycycline for doxy-PEP and 6% for doxy-PrEP (18). A study conducted in the Netherlands reported a similar prevalence of 15% (19). In general, MSM exhibit high willingness but low actual use of doxycycline prophylaxis. Previous studies have shown that more than half of MSM were willing to use doxycycline prophylaxis for STIs prevention (20–22), a proportion substantially higher than the observed utilization proportion. This discrepancy may be due to inadequate awareness of STIs risks and insufficient knowledge regarding doxycycline prophylaxis (21, 23). Additionally, concerns about potential adverse drug effects may deter use. Future efforts should strengthen STIs-related education targeting MSM, improve their knowledge of doxycycline prophylaxis, and promote its timely and appropriate use. This study observed that older participants were less likely to use doxycycline prophylaxis, which may partly contribute to their elevated STIs burden. Evidence from previous studies indicated that older MSM had a higher prevalence of bacterial STIs (24), underscoring the substantial need for targeted prevention within this population. Cultural factors may also play a role. Influenced by traditional norms, older individuals may hold misconceptions or stigmatizing attitudes toward STIs, leading to limited awareness and reduced uptake of doxycycline prophylaxis. These findings highlight the importance of developing tailored interventions targeting older MSM to enhance STIs prevention awareness and promote appropriate use of doxycycline prophylaxis, which could help reduce STIs transmission in this group.

In the present study, a considerable proportion of participants reported engaging in high-risk sexual behaviors, including multiple sexual partners, casual sex, commercial sex, and group sex, indicating a heightened risk of STIs infection. Risk perception serves as a key determinant of preventive behavior. Consistent with this, participants who had casual or commercial sex in the past six months were more likely to use doxycycline prophylaxis for STIs prevention. However, no significant difference in doxycycline use was observed among those reporting multiple sexual partners or group sex, suggesting insufficient recognition of the risks associated with these behaviors. This finding underscores the need for targeted educational interventions to enhance STIs risk awareness and promote evidence-based prevention strategies among MSM. Furthermore, since doxycycline prophylaxis in China can only be obtained through a physician's prescription, limited accessibility may further constrain its uptake. Future research and policy efforts should explore strategies to improve access to doxycycline prophylaxis.

Consistent condom use remains one of the most effective measures for preventing STIs. However, due to limited risk awareness and the pursuit of sexual pleasure, consistent and correct condom use among MSM remains relatively low (25, 26). In this study, only 51.5% of participants reported consistent condoms use during sex, suggesting an elevated risk of STIs transmission. HIV PrEP/PEP, as innovative biomedical prevention approaches, have demonstrated high efficacy in HIV prevention (27, 28). Furthermore, they have been increasingly promoted among MSM in China in recent years (29). In our study, 35.6% of participants reported having used HIV PrEP/PEP, and this group showed a higher prevalence of doxycycline prophylaxis use. This association may reflect stronger health awareness and greater preventive motivation among individuals who engage in HIV PrEP/PEP. Alternatively, evidence suggested that HIV PrEP/PEP use may lead to increased high-risk sexual behaviors among MSM, thereby raising their STIs risk and driving demand for additional preventive measures such as doxycycline prophylaxis (30, 31). Unlike HIV PrEP/PEP, doxycycline is an antibiotic and poses potential risks of antimicrobial resistance (32). Evidence has shown that doxy-PEP may promote not only tetracycline resistance but also cross-resistance to other antimicrobial agents (33). From a public health perspective, doxycycline prophylaxis may not be suitable for large-scale implementation and should be administered in a selective and targeted manner (34). Its implementation should be accompanied by antimicrobial stewardship and continuous surveillance of resistance patterns.

This study has several limitations. First, as a cross-sectional survey, the findings can only identify factors associated with doxycycline prophylaxis use among MSM, and cannot establish causal relationships. Second, participants were recruited through convenience sampling, which may limit the representativeness of the sample. Thus, the generalizability of the findings should be interpreted with caution. Future studies should adopt random sampling strategies to enhance external validity. Third, as data were collected through an online questionnaire, potential privacy concerns and stigma may have introduced social desirability bias.

## Conclusions

Doxycycline prophylaxis utilization among MSM in China remains at a relatively low level. Older MSM reported a lower usage proportion, whereas individuals engaging in casual or commercial sex were more likely to use doxycycline prophylaxis. These patterns may reflect inadequate awareness of STIs risks, insufficient knowledge of doxycycline prophylaxis, and restricted accessibility in China. Targeted educational interventions are urgently needed to enhance STIs prevention awareness among MSM. Besides, future research could integrate qualitative insights to explore barriers and facilitators of doxycycline use, developing more effective and acceptable STIs prevention strategies.

## Data Availability

The original contributions presented in the study are included in the article/supplementary material, further inquiries can be directed to the corresponding author.

## References

[1] WHO. Sexually Transmitted Infections (STIs). (2025). Available online at: https://www.who.int/news-room/fact-sheets/detail/sexually-transmitted-infections-(stis) (Accessed May 29, 2025)

[2] Philibert P Khiri H Pénaranda G Camus C Drogoul MP Halfon P. High prevalence of asymptomatic sexually transmitted infections among men who have sex with men. J Clin Med. (2014) 3:1386–91. doi: 10.3390/jcm3041386 26237608 PMC4470190

[3] WHO. Global and Regional STI Estimates. (2025). Available online at: https://www.who.int/data/gho/data/themes/topics/global-and-regional-sti-estimates (Accessed July 25, 2025).

[4] Jian H Lu WJ Chen ZW Liang SQ Yue XL Li J . Prevalence and trends of Chlamydia trachomatis infection in female sex workers and men who have sex with men in China: a systematic review and meta-analysis. BMC Public Health. (2024) 24:1579. doi: 10.1186/s12889-024-18804-3 38867197 PMC11170796

[5] Xu W Li H Zhao P Wang J Liang P Wang C. Trends of chlamydia and gonorrhea infections by anatomic sites among men who have sex with men in south China: a surveillance analysis from 2018 to 2022. BMC Public Health. (2024) 24:2484. doi: 10.1186/s12889-024-19987-5 39267000 PMC11391739

[6] Lu WJ Jian H Wu YL Zhu WQ Yue XL Fu GF . Prevalence and trend of gonorrhea in female sex workers and men having sex with men in China: a systematic review and meta-analysis. Public Health. (2023) 221:106–15. doi: 10.1016/j.puhe.2023.06.010 37441994

[7] Su R Liu Y Shan D Li P Ge L Li D. Prevalence of HIV/syphilis co-infection among men who have sex with men in China: a systematic review and meta-analysis. BMC Public Health. (2025) 25:1297. doi: 10.1186/s12889-025-22499-5 40197258 PMC11974192

[8] Huang Z Wang M Fu L Fang Y Hao J Tao F . Intervention to increase condom use and HIV testing among men who have sex with men in China: a meta-analysis. AIDS Res Hum Retroviruses. (2013) 29:441–8. doi: 10.1089/aid.2012.0151 23083341

[9] Chow EP Wilson DP Zhang L. Patterns of condom use among men who have sex with men in China: a systematic review and meta-analysis. AIDS Behav. (2012) 16:653–63. doi: 10.1007/s10461-011-9935-9 21461948

[10] Xu W Zheng L Liu Y Zheng Y. Sexual sensation seeking, sexual compulsivity, and high-risk sexual behaviours among gay/bisexual men in Southwest China. AIDS Care. (2016) 28:1138–44. doi: 10.1080/09540121.2016.1153587 26924809

[11] Traeger MW Leyden WA Volk JE Silverberg MJ Horberg MA Davis TL . Doxycycline postexposure prophylaxis and bacterial sexually transmitted infections among individuals using HIV preexposure prophylaxis. JAMA Intern Med. (2025) 185:273–81. doi: 10.1001/jamainternmed.2024.7186 39761062 PMC11877173

[12] Parker AM Chang JJ Chen L King LM Mccoy SI Lewnard JA . Bacterial sexually transmitted infections and related antibiotic use among individuals eligible for doxycycline post-exposure prophylaxis in the United States. Nat Commun. (2025) 16:9206. doi: 10.1038/s41467-025-64261-w 41102210 PMC12532492

[13] Bachmann LH Barbee LA Chan P Reno H Workowski KA Hoover K . CDC clinical guidelines on the use of doxycycline postexposure prophylaxis for bacterial sexually transmitted infection prevention, United States, 2024. MMWR Recomm Rep. (2024) 73:1–8. doi: 10.15585/mmwr.rr7302a1 38833414 PMC11166373

[14] Saunders J Deering J Dewsnap C Drayton R Gilmore J Grant A . British Association for Sexual Health and HIV (BASHH) UK national guideline for the use of doxycycline post-exposure prophylaxis (DoxyPEP) for the prevention of syphilis. Int J STD AIDS. (2025) 2025:9564624251352053. doi: 10.1177/09564624251352053 40505019

[15] Werner RN Schmidt AJ Potthoff A Spornraft-Ragaller P Brockmeyer NH German STIS. Position statement of the German STI Society on the prophylactic use of doxycycline to prevent STIs (Doxy-PEP, Doxy-PrEP). J Dtsch Dermatol Ges. (2024) 22:466–78. doi: 10.1111/ddg.15282 38123738

[16] Cornelisse VJ Riley B Medland NA. Australian consensus statement on doxycycline post-exposure prophylaxis (doxy-PEP) for the prevention of syphilis, chlamydia and gonorrhoea among gay, bisexual and other men who have sex with men. Med J Aust. (2024) 220:381–6. doi: 10.5694/mja2.52258 38479437

[17] Chen YT Lin KY Sun HY Huang YS Liu WD Chuang YC . Awareness and willingness toward doxycycline post-exposure prophylaxis use for bacterial sexually transmitted infections among men who have sex with men. Sex Health. (2025) 22:SH24136. doi: 10.1071/SH24136 40418726

[18] Hornuss D Mathe P Usadel S Zimmermann S Muller M Rieg S. Already current practice? A snapshot survey on doxycycline use for prevention of sexually transmitted infections in parts of the German MSM community. Infection. (2023) 51:1831–4. doi: 10.1007/s15010-023-02086-9 37608042 PMC10665247

[19] Teker B Hoornenborg E Schim Van Der Loeff MF Boyd A Heijne JC Prins M . Emergent informal use of doxycycline post- and pre-exposure prophylaxis among men who have sex with men and transgender and gender diverse people, the Netherlands, 2024. Euro Surveill. (2025) 30:2400707. doi: 10.2807/1560-7917.ES.2025.30.26.2400707 40613130 PMC12231377

[20] Wagner L Boesecke C Baumgarten A Scholten S Schellberg S Hoffmann C . Is doxycycline post-exposure prophylaxis being utilised in Germany? Insights from an online survey among German men who have sex with men. Infection. (2025) 53:61–70. doi: 10.1007/s15010-024-02321-x 39042326 PMC11825561

[21] Bui HTM Adamson PC Klausner JD Le GM Gorbach PM. Doxycycline prophylaxis for bacterial sexually transmitted infection prevention in Vietnam: awareness, attitudes and willingness to use among men who have sex with men using HIV-PrEP. Sex Transm Infect. (2025) 101:367–73. doi: 10.1101/2024.12.02.24318296 39915103 PMC12325689

[22] Liang P Zhao P Huang S Wang C. Willingness to accept doxycycline post-exposure prophylaxis for bacterial stis prevention among men who have sex with men in Southern China: a cross-sectional analysis. BMC Infect Dis. (2025) 25:911. doi: 10.1186/s12879-025-11290-x 40646493 PMC12247278

[23] Vanbaelen T Rotsaert A De Baetselier I Platteau T Hensen B Reyniers T . Doxycycline post-exposure prophylaxis among men who have sex with men and transgender women in Belgium: awareness, use and antimicrobial resistance concerns in a cross-sectional online survey. Sex Transm Infect. (2025) 101:34–40. doi: 10.1136/sextrans-2024-056261 39209541 PMC11877044

[24] Wu TY Lin KY Su LH Sun HY Huang YS Liu WD . Sexually transmitted coinfections among at-risk HIV-positive MSM: implications for optimal preemptive treatment. Front Med (Lausanne). (2024) 11:1328589. doi: 10.3389/fmed.2024.1328589 38560383 PMC10978595

[25] Huang Y Yu B Jia P Wang Z Yang S Tian C . Association between psychological factors and condom use with regular and nonregular male sexual partners among Chinese MSM: a quantitative study based on the health belief model. Biomed Res Int. (2020) 2020:5807162. doi: 10.1155/2020/5807162 33062685 PMC7539081

[26] Wray TB Monti PM. Characteristics of sex events, partners, and motivations and their associations with HIV-risk behavior in a daily diary study of high-risk men who have sex with men (MSM). AIDS Behav. (2020) 24:1851–64. doi: 10.1007/s10461-019-02760-w 31832855 PMC7228849

[27] Desai M Field N Grant R Mccormack S. Recent advances in pre-exposure prophylaxis for HIV. BMJ. (2017) 359:j5011. doi: 10.1136/bmj.j5011 29229609 PMC6020995

[28] Irvine C Egan KJ Shubber Z Van Rompay KK Beanland RL Ford N. Efficacy of HIV postexposure prophylaxis: systematic review and meta-analysis of nonhuman primate studies. Clin Infect Dis. (2015) 60:S165–9. doi: 10.1093/cid/civ069 25972498

[29] Guidelines for AIDS Prevention Among Men who have Sex with Men. (2016). Available online at: https://ncaids.chinacdc.cn/2018zlxz/201611/t20161117_135765.htm (Accessed September 13, 2016)

[30] Su R Liu Y Li P Ge L Liao M Fu Y . Utilization of post-exposure prophylaxis potentially contributed to the changes of risk behaviors among men who have sex with men in China. Front Public Health. (2024) 12:1364913. doi: 10.3389/fpubh.2024.1364913 38651127 PMC11033407

[31] Milam J Jain S Dubé MP Daar ES Sun X Corado K . Sexual risk compensation in a pre-exposure prophylaxis demonstration study among individuals at risk of HIV. J Acquir Immune Defic Syndr. (2019) 80:e9–e13. doi: 10.1097/QAI.0000000000001885 30334877 PMC6289757

[32] Pitt-Kendall R Sun S Hughes S Merrick R Donaldson H Rayment M . Investigating the cause of increased tetracycline-resistant Neisseria gonorrhoeae in England, 2016-20. J Antimicrob Chemother. (2024) 79:1060–8. doi: 10.1093/jac/dkae073 38517444 PMC11062939

[33] Gestels Z Manoharan-Basil SS Kenyon C. Doxycycline post exposure prophylaxis could select for cross-resistance to other antimicrobials in various pathogens: an in silico analysis. Int J STD AIDS. (2023) 34:962–8. doi: 10.1177/09564624231190108 37466467

[34] Mardh O Plachouras D. Using doxycycline for prophylaxis of bacterial sexually transmitted infections: considerations for the European Union and European Economic Area. Euro Surveill. (2023) 28:2300621. doi: 10.2807/1560-7917.ES.2023.28.46.2300621 37971658 PMC10655202

